# If It Works, Don’t Touch It? A Cell-Based Approach to Studying 2-[^18^F]FDG Metabolism

**DOI:** 10.3390/ph14090910

**Published:** 2021-09-09

**Authors:** Eva-Maria Klebermass, Mahshid Mahmudi, Barbara Katharina Geist, Verena Pichler, Chrysoula Vraka, Theresa Balber, Anne Miller, Arvand Haschemi, Helmut Viernstein, Nataliya Rohr-Udilova, Marcus Hacker, Markus Mitterhauser

**Affiliations:** 1 Division of Nuclear Medicine, Department of Biomedical Imaging and Image-Guided Therapy, Medical University of Vienna, 1090 Vienna, Austria; eva-maria.klebermass@meduniwien.ac.at (E.-M.K.); mahshid.mahmudi@fhwn.ac.at (M.M.); barbara.geist@meduniwien.ac.at (B.K.G.); chrysoula.vraka@meduniwien.ac.at (C.V.); theresa.balber@lbiad.lbg.ac.at (T.B.); marcus.hacker@meduniwien.ac.at (M.H.); 2 Division of Pharmaceutical Technology and Biopharmaceutics, Department of Pharmaceutical Sciences, University of Vienna, 1090 Vienna, Austria; helmut.viernstein@univie.ac.at; 3 Division of Pharmaceutical Chemistry, Department of Pharmaceutical Sciences, University of Vienna, 1090 Vienna, Austria; verena.pichler@univie.ac.at; 4 Ludwig Boltzmann Institute Applied Diagnostics, 1090 Vienna, Austria; 5 Department of Laboratory Medicine, Medical University of Vienna, 1090 Vienna, Austria; annelisabethmiller@googlemail.com (A.M.); arvand.haschemi@meduniwien.ac.at (A.H.); 6 Division of Gastroenterology and Hepatology, Department of Internal Medicine III, Medical University of Vienna, 1090 Vienna, Austria; nataliya.rohr-udilova@meduniwien.ac.at

**Keywords:** 2-[^18^F]FDG, 2-[^18^F]FDG metabolism, PET-tracer metabolism, molecular imaging, cancer metabolism

## Abstract

The glucose derivative 2-[^18^F]fluoro-2-deoxy-D-glucose (2-[^18^F]FDG) is still the most used radiotracer for positron emission tomography, as it visualizes glucose utilization and energy demand. In general, 2-[^18^F]FDG is said to be trapped intracellularly as 2-[^18^F]FDG-6-phosphate, which cannot be further metabolized. However, increasingly, this dogma is being questioned because of publications showing metabolism beyond 2-[^18^F]FDG-6-phosphate and even postulating 2-[^18^F]FDG imaging to depend on the enzyme hexose-6-phosphate dehydrogenase in the endoplasmic reticulum. Therefore, we aimed to study 2-[^18^F]FDG metabolism in the human cancer cell lines HT1080, HT29 and Huh7 applying HPLC. We then compared 2-[^18^F]FDG metabolism with intracellular tracer accumulation, efflux and the cells’ metabolic state and used a graphical Gaussian model to visualize metabolic patterns. The extent of 2-[^18^F]FDG metabolism varied considerably, dependent on the cell line, and was significantly enhanced by glucose withdrawal. However, the metabolic pattern was quite conserved. The most important radiometabolites beyond 2-[^18^F]FDG-6-phosphate were 2-[^18^F]FDMannose-6-phosphate, 2-[^18^F]FDG-1,6-bisphosphate and 2-[^18^F]FD-phosphogluconolactone. Enhanced radiometabolite formation under glucose reduction was accompanied by reduced efflux and mirrored the cells’ metabolic switch as assessed via extracellular lactate levels. We conclude that there can be considerable metabolism beyond 2-[^18^F]FDG-6-phosphate in cancer cell lines and a comprehensive understanding of 2-[^18^F]FDG metabolism might help to improve cancer research and tumor diagnosis.

## 1. Introduction

The radioactively labelled glucose derivative 2-[^18^F]fluoro-2-deoxy-D-glucose (2-[^18^F]FDG) is still the most used tracer in positron emission tomography (PET) [1,2]. In principle, this tracer is said to visualize the so-called Warburg Effect, which is described as a shift towards glycolysis and lactate overproduction despite a normoxic environment [2,3]. This ineffective way to generate energy causes cells to consume more glucose [3]. As this is a major feature of most tumors, PET imaging with 2-[^18^F]FDG, together with computer tomography (CT), is an indispensable tool for the precise localization and simultaneous metabolic characterization of malignant lesions. Aside from cancer diagnosis or staging, 2-[^18^F]FDG is used for the assessment of myocardial viability and the characterization and imaging of neurodegenerative diseases [1,2]. Firstly, higher glucose utilization might also occur naturally in healthy tissues such as brain (potentially complicating diagnosis), or at sites of inflammation (causing false-positive results in cancer diagnosis) [1,2]. Moreover, because of their metabolic makeup, some cancers by nature show low 2-[^18^F]FDG avidity [4], calling for more specific, receptor-based tracers.

Although 2-[^18^F]FDG has been widely used and comprehensively studied, its exact mode of action remains unclear. In a study by Li et al. with mice bearing peritoneal carcinomatosis and subcutaneous tumors, 2-[^18^F]FDG uptake was presented as a readout for hypoxia (anaerobic glycolysis) rather than depicting the Warburg Effect (aerobic glycolysis) [5]. In other studies with non-small cell lung cancer patients [6,7], where 2-[^18^F]FDG PET was combined with [13C]glucose tracing, enhanced 2-[^18^F]FDG uptake did not correlate to glycolytic metabolism, but to respiration [6] and to the proliferation index [7]. The latter study hence suggests 2-[^18^F]FDG PET-signal to be a readout for the use of glucose in pathways other than glycolysis.

Moreover, 2-[^18^F]FDG’s own intracellular “standstill” after phosphorylation is being questioned. In general, 2-[^18^F]FDG is taken up via glucose transporters, but unlike glucose it is said to be trapped as 2-[^18^F]FDG-6-phosphate (2-[^18^F]FDG-6-P) after its phosphorylation by hexokinase (“metabolic trapping”). In contrast to glucose-6-phosphate, the isomerization to an analogue of fructose-6-phosphate, which would be necessary for further processing through the glycolytic pathway, is hindered by the fluoride in position 2 [1,2]. Moreover, 2-[^18^F]FDG PET-signal quantification nowadays still builds on the assumption that 2-[^18^F]FDG-6-P is the only metabolite. The refined Sokoloff compartment model used for quantification lists three or four rate constants, describing the transport of 2-[^18^F]FDG from blood pool to tissue, the redistribution of 2-[^18^F]FDG into blood, phosphorylation leading to intracellular retention and occasionally the dephosphorylation of 2-[^18^F]FDG-6-P by glucose-6-phosphatase (G6Pase) [8], as seen in liver tissue due to gluconeogenesis [9]. However, although 2-[^18^F]FDG cannot be processed through glycolysis, it can still be metabolized beyond 2-[^18^F]FDG-6-P [10]. Notably, the glucose analogue seems to be shuttled into glycogen metabolism or the pentose phosphate pathway (PPP) [11]. Its metabolites have mostly been analyzed using its non-radioactive analogue 2-deoxy-2-fluoro-D-glucose and 19F-NMR [11,12,13]. However, this approach demands the application of vast amounts of non-radioactive FDG, which could—due to mass effects—result in a different outcome when compared to trace amounts used with 2-[^18^F]FDG. Other groups that tested metabolism of the radioactive form 2-[^18^F]FDG in xenograft mice and rats detected fewer metabolites, potentially due to sensitivity limitations of their HPLC system [14,15].

Recently, research on 2-[^18^F]FDG metabolism has gained new momentum with publications describing intracellular 2-[^18^F]FDG accumulation as specifically being a function of hexose-6-phosphate dehydrogenase (H6PD), the first enzyme of the oxidative branch of the PPP located in the endoplasmic reticulum (ER) [16,17].

Although 2-[^18^F]FDG is increasingly replaced by more specific PET-tracers, it is still an indispensable tool in nuclear medicine. While the simplistic model of the tracer’s intracellular trapping might be sufficient for some clinical questions, an in-depth understanding of its intracellular fate could improve basic cancer research or cancer diagnosis. We hypothesized that 2-[^18^F]FDG metabolism would differ significantly in cancer cell lines of different tumor entities, as well as under different glucose conditions. Therefore we studied the 2-[^18^F]FDG metabolism in human cell lines of fibrosarcoma (HT1080), colorectal adenocarcinoma (HT29) and hepatocellular carcinoma (Huh7). We aimed to elucidate metabolic patterns and pathways involved in the tracer’s metabolism to create a basis for a better understanding of cancer-relevant 2-[^18^F]FDG accumulation and efflux.

## 2. Results

### 2.1. Enzymatic In Vitro Preparation and Verification of Radiometabolites

**Figure 1:**
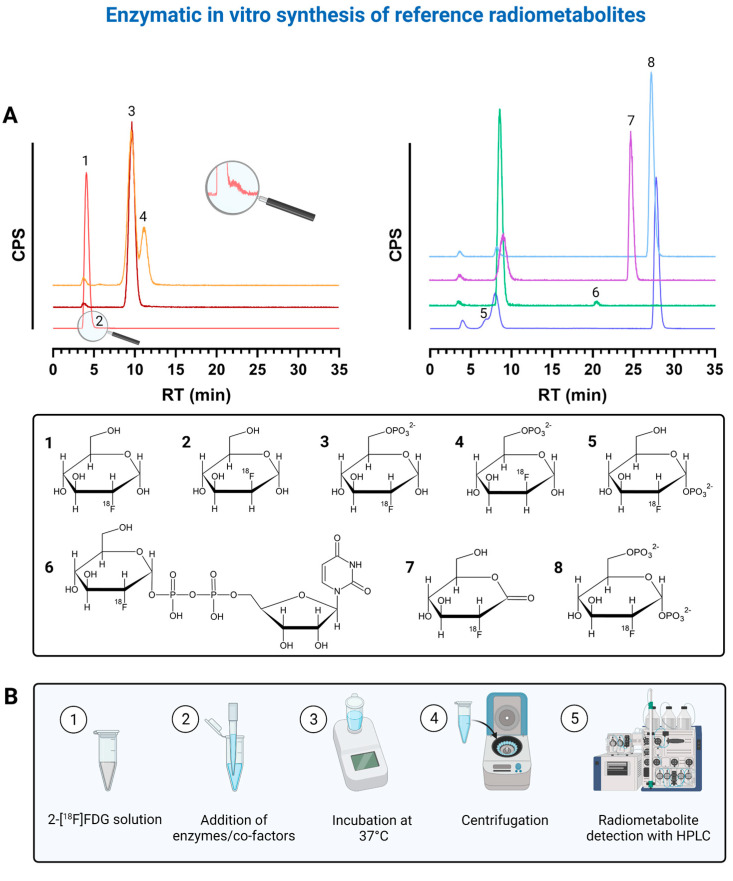
(**A**) shows representative chromatograms of the reference radiometabolites that were prepared via enzymatic in vitro synthesis and corresponding chemical structures. Each color represents the chromatogram of a different reference compound. 1 = 2-[^18^F]FDG, 2 = 2-[^18^F]FDM, 3 = 2-[^18^F]FDG-6-P, 4 = 2-[^18^F]FDM-6-P, 5 = 2-[^18^F]FDG-1-P, 6 = UDP-2-[^18^F]FDG, 7 = 2-[^18^F]FD-PGL, 8 = 2-[^18^F]FDG-1,6-P_2_. (**B**) shows the corresponding workflow of enzymatic in vitro synthesis of reference radiometabolites. Created with BioRender.com (accessed on 4 September 2021) based on HPLC raw data and graphs from Prism 7.03 software.

To properly identify the radiometabolites of 2-[^18^F]FDG, we started to synthesize potential radiometabolites via enzymatic in vitro preparation. After adapting the HPLC assay by Rokka et al. [18] to our system in terms of gradient profile and run time (Table S1), we could verify the identity of the following radiometabolites: 2-[^18^F]FDG-1-phosphate (2-[^18^F]FDG-1-P, retention time (RT)~7 min), 2-[^18^F]FDG-6-P (RT~9.5 min), 2-[^18^F]FDMannose-6-phosphate (2-[^18^F]FDM-6-P, RT~12 min), uridine diphosphate-2-[^18^F]FDG (UDP-2-[^18^F]FDG, RT~21 min), 2-[^18^F]FD-phosphogluconolactone (2-[^18^F]FD-PGL, RT~25 min) and 2-[^18^F]FDG-1,6-bisphosphate (2-[^18^F]FDG-1,6-P_2_, RT~28 min). Figure 1A shows representative chromatograms of the reference radiometabolites and corresponding chemical structures, while Figure 1B shows the workflow of the enzymatic in vitro synthesis of reference radiometabolites.

We could not identify the radiometabolites at ~19.5, ~22, ~23.5, ~27 and ~29.5 min. However, the radiometabolites at ~22, ~23.5 and ~27 min never exceeded 0.1, 0.3 and 0.05%, respectively. 2-[^18^F]FDG-6-P and 2-[^18^F]FDM-6-P were not fully separable despite HPLC optimization. Rokka et al. [18] reported that UDP-2-[^18^F]FDG was not synthesized because when adding phosphoglucomutase (PGM), the conversion of 2-[^18^F]FDG-6-P to 2-[^18^F]FDG-1,6-P_2_ via 2-[^18^F]FDG-1-P is always favored over UDP-2-[^18^F]FDG formation in the presence of hexokinase. Therefore, we filtered our 2-[^18^F]FDG-6-P solution containing hexokinase through a 30 kDa filter prior to the addition of PGM, uridine triphosphate (UTP) and UDP-glucose-pyrophosphorylase (UGPase). However, when we additionally added glucose-1,6-bisphosphate, ethylenediaminetetraacetic acid (EDTA) and bovine serum albumin (BSA) to the solution after filtering, with or without UTP and UGPase, 2-[^18^F]FDG-1,6-P_2_ was always the main metabolite after ~2.5 h. UDP-2-[^18^F]FDG was not produced. Interestingly, when we did not introduce any additional compounds to the filtrate mixed with UTP and UGPase, 2-[^18^F]FDG-1,6-P_2_ was not or was only merely produced and we finally saw a metabolite at ~21 min, namely UDP-2-[^18^F]FDG (Figure 1A). We observed that 2-[^18^F]FDG-1,6-P_2_, a natural intermediate of the conversion of 2-[^18^F]FDG-6-P to 2-[^18^F]FDG-1-P, is also produced in the absence of hexokinase. Adding glucose-1,6-bisphosphate, EDTA and BSA seemed to shift the equilibrium towards the intermediate 2-[^18^F]FDG-1,6-P_2_ and UDP-2-[^18^F]FDG cannot be built. We once also detected an additional peak at ~7 min after around 2 h when synthesizing 2-[^18^F]FDG-1,6-P_2_, which should be 2-[^18^F]FDG-1-P (Figure 1A).

Furthermore, we identified the peak at ~12 min as 2-[^18^F]FDM-6-P (Figure 1A). The injection of pure 2-[^18^F]FDG showed an additional peak with an RT of ~6 min. This peak should be 2-[^18^F]FDMannose, a by-product of 2-[^18^F]FDG production that results from epimerization during alkaline hydrolysis (Figure 1A) [19]. Furthermore, we synthesized 2-[^18^F]FD-PGL using two different commercially available, recombinant glucose-6-phosphate dehydrogenases (G6PDs) (Figure 1A). As 2-[^18^F]FD-PGL is not stable [20], the peak at ~25 min can also be the decomposition product 2-[^18^F]FD-phosphogluconate or a mixture of both.

Overall, we verified the identity of three more radiometabolites (2-[^18^F]FDG-1-P, 2-[^18^F]FDM-6-P and UDP-2-[^18^F]FDG) with enzymatic in vitro synthesis as compared to Rokka et al. [18], facilitating future analyses of 2-[^18^F]FDG metabolism.

### 2.2. Intracellular Accumulation and Metabolism of 2-[^18^F]FDG in HT1080 (Fibrosarcoma), HT29 (Colorectal Adenocarcinoma) and Huh7 (Hepatocellular Carcinoma)

**Figure 2:**
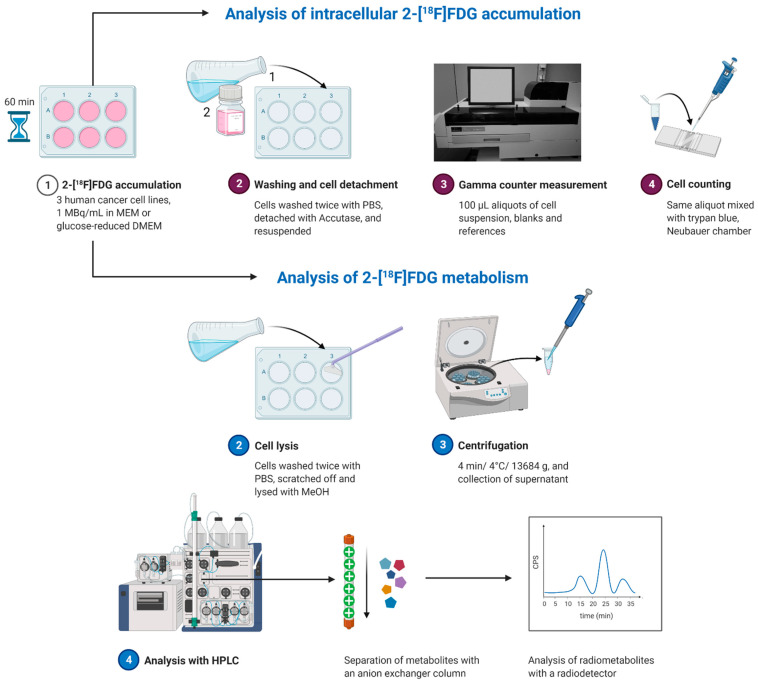
Workflow of experiments determining intracellular 2-[^18^F]FDG accumulation and metabolism. Adapted from “Protein Purification by Size-Exclusion Chromatography” by BioRender.com (accessed on 4 September 2021). Retrieved from https://app.biorender.com/biorender-templates.

**Figure 3:**
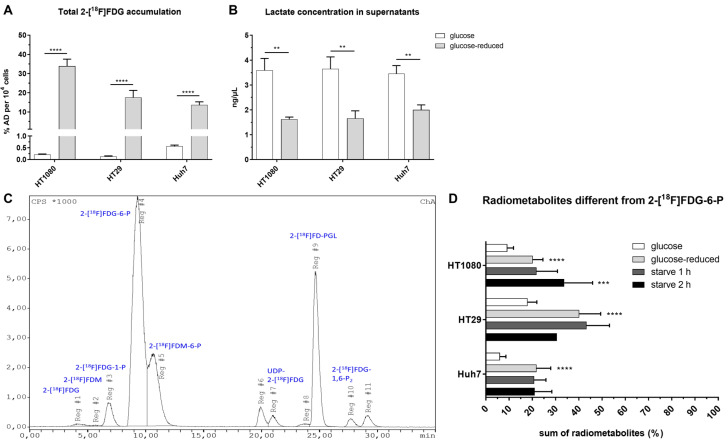
Total intracellular 2-[^18^F]FDG accumulation after 1 h (**A**) and lactate concentration in the supernatants at the end of the experiment (** *p* ≤ 0.01) (**B**). (**C**) shows a representative image of the radiometabolite pattern under glucose-reduced conditions in HT29 after 1 h and (**D**) the sum of radiometabolites other than 2-[^18^F]FDG-6-P after 1 h in % (**** *p* ≤ 0.0001, *** *p* ≤ 0.001 compared to glucose conditions). Conditions: glucose = 1.13 g/L, glucose-reduced = 0.13 g/L, starve = glucose-reduced medium 1 or 2 h prior to 2-[^18^F]FDG addition. If not visible, the error bars are within the margin of the symbols.

2-[^18^F]FDG was applied to the cells either under glucose (1.13 g/L), glucose-reduced (0.13 g/L) or starving conditions (glucose-reduced medium 1 or 2 h prior to 2-[^18^F]FDG addition). Figure 2 summarizes the workflow of experiments determining 2-[^18^F]FDG accumulation and 2-[^18^F]FDG metabolism in cancer cells. Under glucose conditions, the highest 2-[^18^F]FDG accumulation in % applied dose (% AD) per 10^4^ cells after 1 h was seen in Huh7 (0.57 ± 0.04%), followed by HT1080 (0.22 ± 0.01%) and HT29 (0.13 ± 0.02%) (Figure 3A). Contrary to this, under glucose-reduced conditions, HT29 and Huh7 showed similar accumulation (18 ± 4% and 14 ± 2%), while there was significantly higher 2-[^18^F]FDG accumulation per 10^4^ cells in HT1080 (34 ± 4%) (Figure 3A). 

Analysis of lactate concentration in supernatants after the experiments showed that extracellular lactate was more than twice as high for HT29 and HT1080 under glucose conditions (HT29: 3.7 ± 0.5% ng/µL; HT1080: 3.6 ± 0.5% ng/µL) compared to glucose-reduced conditions (HT29: 1.7 ± 0.3% ng/µL; HT1080: 1.6 ± 0.1% ng/µL). In Huh7, the difference between glucose and glucose-reduced conditions was slightly smaller with 3.5 ± 0.3% vs. 2.0 ± 0.2% ng/µL (Figure 3B). These data indicate a metabolic switch in all three cell lines following glucose reduction. 

Overall, we could detect up to 12 radiometabolites in HPLC when stopping 2-[^18^F]FDG accumulation after 1 h. Figure 3C shows a representative image of the metabolic pattern in HT29 using glucose-reduced medium, Figure 3D summarizes the extent of metabolism beyond 2-[^18^F]FDG and 2-[^18^F]FDG-6-P in the three cell lines. Except for the metabolite at ~27 min, which was only observed in HT1080, all radiometabolites were detected in all three cell lines. Under glucose conditions, HT29 showed the highest levels of radiometabolites other than 2-[^18^F]FDG-6-P after 1 h with 18 ± 4%, followed by HT1080 (9 ± 3%) and Huh7 (6 ± 3%). It was the same cell line that showed the highest radiometabolite formation under glucose-reduced conditions with 40 ± 9%, while glucose reduction led to similar results in HT1080 and Huh7 (20 ± 4% and 22 ± 6%). Starving did not significantly enhance metabolism beyond 2-[^18^F]FDG in Huh7 or HT29 compared to glucose-reduced conditions. However, 2 h starving significantly enhanced radiometabolite formation in HT1080 to 34 ± 12% (*p* ≤ 0.001 compared to glucose conditions).

In summary, glucose withdrawal led to increased intracellular 2-[^18^F]FDG accumulation and enhanced radiometabolite formation dependent on the cell line.

Table 1 gives a more detailed overview on the change of radiometabolite formation dependent on the glucose concentration. In general, the most prominent radiometabolites rising when glucose in the medium was reduced were 2-[^18^F]FDG-1-P, 2-[^18^F]FDM-6-P and 2-[^18^F]FD-PGL. In contrast to that, besides parent 2-[^18^F]FDG and 2-[^18^F]FDG-6-P, levels of 2-[^18^F]FDG-1,6-P_2_ decreased under glucose deprivation. HT29 was the only cell line with noteworthy radiometabolite formation beyond 2-[^18^F]FDG-6-P under glucose conditions and also the cell line with the highest 2-[^18^F]FD-PGL formation under glucose (3 ± 1%) and glucose-reduced (15 ± 7%) conditions.

**Figure 4:**
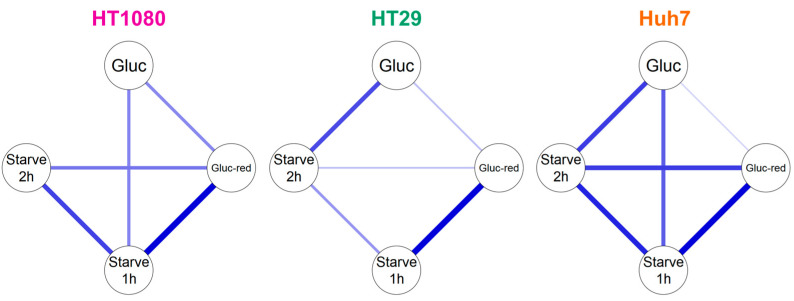
Graphical Gaussian model of 2-[^18^F]FDG metabolism in HT1080, HT29 and Huh7. Blue lines show positive correlations. The thicker the line, the higher the correlation.

Building on Table 1, we analyzed our HPLC data with a graphical Gaussian model to identify and illustrate relevant correlations between radiometabolite formation under different glucose conditions (Figure 4). In accordance with Table 1, the graphical Gaussian model demonstrates that the cell lines handle 2-[^18^F]FDG differently dependent on glucose concentration. Overall, positive partial correlations between radiometabolite formation under different conditions were particularly pronounced in Huh7, while they were the least pronounced in HT29. However, the highest positive partial correlation was observed between glucose-reduced (gluc-red) and starve (1 h) conditions in HT29, with a value of 0.73.

### 2.3. Efflux of Radioactivity in HT1080 (Fibrosarcoma) and HT29 (Colorectal Adenocarcinoma)

**Figure 5:**
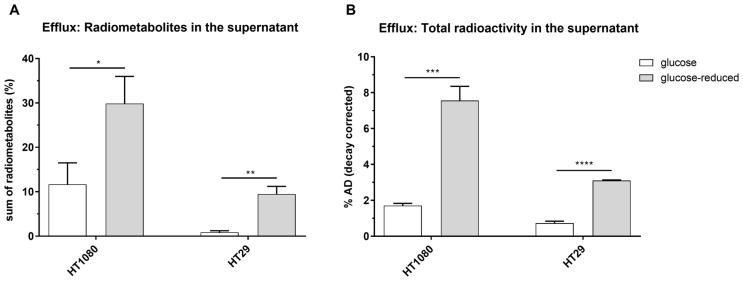
Sum of radiometabolites (%) detected with HPLC (**A**) and total radioactivity in % applied dose (% AD) in supernatants of HT1080 and HT29 1 h after change to non-radioactive medium (**B**) (* *p* ≤ 0.05, ** *p* ≤ 0.01, *** *p* ≤ 0.001 and **** *p* ≤ 0.0001). Conditions: glucose = 1.13 g/L, glucose-reduced = 0.13 g/L.

In general, as expected, 2-[^18^F]FDG was the most prominent compound when analyzing the supernatant with HPLC, 1 h after medium change. However, 2-[^18^F]FDM and 2-[^18^F]FDG-6-P could be detected additionally under glucose conditions and 2-[^18^F]FDM, 2-[^18^F]FDG-1-P, 2-[^18^F]FDG-6-P and 2-[^18^F]FD-PGL when glucose content was reduced. In HT1080 cells, levels of extracellular radiometabolites were higher than in HT29, both under glucose or glucose-reduced conditions (Figure 5A). Under glucose-reduced conditions, levels of extracellular radiometabolites were significantly elevated in both cell lines 1 h after medium change.

Quantifying radioactivity in the supernatant 1 h after the change to non-radioactive medium, decay-corrected amounts were 1.7 ± 0.1% AD (glucose) and 7.6 ± 0.8% AD (glucose-reduced) for HT1080 and 0.7 ± 0.1% AD (glucose) and 3.1 ± 0.3% AD (glucose-reduced) for HT29 (Figure 5B).

In the lactate dehydrogenase (LDH) assay, no change of extracellular LDH activity was seen in the supernatants between time-point 1 (before medium change) and time-point 2 (1 h after medium change) (Figure S1), indicating no leakage of cell membranes.

## 3. Discussion

Rokka et al. [18] presented a suitable radio-HPLC assay for the analysis of 2-[^18^F]FDG metabolism identifying the radiometabolites 2-[^18^F]FDG-6-P, 2-[^18^F]FDG-1,6-P_2_ and 2-[^18^F]FD-PGL. Building on their assay, we additionally verified the formation of the radiometabolites 2-[^18^F]FDG-1-P, 2-[^18^F]FDM-6-P and UDP-2-[^18^F]FDG. In particular, the formation of FDM-6-P was described in several 19F-NMR studies with FDG before [11,12,13,21]. It was postulated that while building an analogue of fructose-6-phosphate is prevented by the fluoride, the formation of 1,2-endiol as an intermediate and lastly FDM-6-P is still possible [22]. However, to the best of our knowledge, we are the first to verify the intracellular formation of 2-[^18^F]FDM-6-P, as well as of the other radiometabolites 2-[^18^F]FDG-1-P and UDP-2-[^18^F]FDG using the radiotracer 2-[^18^F]FDG. Furthermore, despite what is often stated [16,23], 2-[^18^F]FDG-6-P seems to also be processed by G6PD and not only H6PD, at least in a cell-free assay in vitro, as already proposed by Suolinna et al. [14]. 

Looking at the accumulation experiments, glucose reduction to one tenth led to an increase in 2-[^18^F]FDG uptake of around 24 times in Huh7, 130 times in HT29 and 150 times in HT1080, although the same amount of radioactivity was used. The comparatively little difference within Huh7 might be rooted in using MEM for the accumulation experiments under glucose conditions. As Huh7 was cultivated in high-glucose DMEM, the change to MEM already resulted in a large glucose reduction for these cells (4.63 g/L to 1.13 g/L). This might also explain why there was a smaller difference of extracellular lactate concentration between glucose and glucose-reduced conditions in this cell line as compared to HT1080 and HT29. 

Our efflux experiments showed that radiometabolites can be found in the cell culture medium 1 h after change to non-radioactive medium. Although the results of the LDH assay indicate no leakage of cell membranes, the 2-[^18^F]FDG accumulation experiments were generally performed in fully supplemented cell culture medium with inherent LDH activity. Therefore, the background was high and small changes in LDH excretion might therefore be hard to detect. Moreover, the LDH assay determines the activity of the enzyme, not the mere presence of it. Hence, in contrast to HPLC measurements of radiometabolites, the results of this assay are subject to many influences and results should therefore be interpreted with caution. Moreover, up to this date, the only known transporters for sugar-phosphates are located at the membranes of intracellular microsomes [24], making a sugar phosphate transport out of the cell unlikely. Still, extracellular phosphorylated 2-[^18^F]FDG metabolite levels and total radioactivity in the supernatant after the efflux experiment were significantly higher in HT1080, both under glucose and glucose-reduced conditions. Interestingly, the intracellular levels of phosphorylated radiometabolites beyond 2-[^18^F]FDG-6-P were around twice as high in HT29 compared to HT1080 after 1 h (glucose: ~18 vs. ~9%; glucose-reduced or starved: ~40 vs. ~20%). Hence, there is less efflux in the cell line that shows higher metabolism. Cossu et al. suggested that the dephosphorylating enzyme G6Pase and H6PD compete for 2-[^18^F]FDG-6-P in the ER [25]. Thus, enhanced production of 2-[^18^F]FDG-PGL by H6PD would significantly reduce dephosphorylation and subsequent efflux of 2-[^18^F]FDG, which would at least explain higher extracellular 2-[^18^F]FDG levels of HT1080. 

The main focus of this paper, however, was to investigate whether a specific pattern of 2-[^18^F]FDG metabolism could be observed in different human cancer cell lines, under different glucose concentrations. In comparison to Rokka et al. we could in general detect more radiometabolites with HPLC analysis. However, compared to our cell assay, where we applied relatively high activities (1MBq/mL), they analyzed tissue samples of rats and mice [18]. Hence, due to biodistribution and subsequent work steps such as organ removal and processing (homogenization, dilution), radioactivity in their samples was most likely lower. Moreover, we saw the highest amounts of radiometabolites when we reduced glucose concentration in the medium. This set-up cannot be applied in an in vivo experiment. 

Although the three tumor cell lines were derived from very different tumor entities (fibrosarcoma, colorectal adenocarcinoma, hepatocellular carcinoma), which in turn originate from different cell types, the radiometabolites generated were almost identical. Interestingly, we saw differences regarding the extent of radiometabolite formation and the cells’ response to glucose reduction or starving (Table 1), suggesting different 2-[^18^F]FDG handling. This was further supported by our graphical Gaussian model, an established mathematical model for network analysis of biological systems [26,27] that we applied to visualize correlations (Figure 4).

We found that—except for HT29—metabolism beyond 2-[^18^F]FDG-6-P was not pronounced under glucose conditions (baseline). However, the extent of 2-[^18^F]FDG metabolism and especially radiometabolites reflecting glycogen metabolism (2-[^18^F]FDG-1-P) or the PPP (2-[^18^F]FD-PGL) rose under glucose reduction. This fact matches the cells’ metabolic switch under glucose reduction as displayed by reduced extracellular lactate levels. Glucose withdrawal is also known to cause a stress situation for glucose-dependent cancer cells [28,29]. Cancer cells can use glycogen metabolism [28] or the PPP [30]—both interconnecting with key metabolic pathways such as glycolysis—to counterbalance (oxidative) stress and sustain growth. Furthermore, we observed enhanced 2-[^18^F]FDM-6-P formation when glucose content was reduced. Interestingly, McSheehy et al. showed in a study with cold FDG and 5-fluorouracil that generated FDM and its phosphorylated conjugates were representative for the treatment response to 5-fluorouracil in mice [21], emphasizing the potential of analyzing FDG metabolism. 

Although we present in vitro data from established cancer cell lines, we propose that glucose availability could also have substantial influence on the uptake and metabolism of 2-[^18^F]FDG in vivo. While Sprinz et al. postulate that the influence of blood glucose levels on 2-[^18^F]FDG uptake in normal organs and tumors is negligible [31], other publications report false-negative 2-[^18^F]FDG tumor PET-scans due to hyperglycemia [32,33,34]. They also highlight the importance of fasting prior to the PET-scan [32]. A fasting period of at least 4 h is also recommended in the latest version of the tumor PET/CT guidelines of the European Association of Nuclear Medicine [35]. 

Furthermore, on a cellular level, factors like tumor vascularization, lymphatics or competition for nutrients with microenvironmental cells can substantially influence nutrient accessibility for tumor cells [36] and could thus influence 2-[^18^F]FDG handling locally.

## 4. Materials and Methods

### 4.1. General

2-[^18^F]FDG was synthesized in-house in a fully automated cassette-based module (FASTlab, GE Healthcare, Uppsala, Sweden) at the Vienna General Hospital, Austria and was applied to the cells after quality control, formulated for patient use. All chemicals were purchased from Merck KGaA (Darmstadt, Germany) and all consumables or cell culture supplies were bought from known suppliers, such as VWR International (Radnor, PA, USA), Merck KGaA or Thermo Fisher Scientific Inc. (Waltham, MA, USA).

### 4.2. Cell Culture

The human fibrosarcoma cell line HT1080 was kindly provided by the Institute of Inorganic Chemistry, University of Vienna. HT1080 was grown in MEM with the addition of 10% FBS and 2 mM L-glutamine. The human colorectal adenocarcinoma cell line HT29 was a generous gift from the Department of Pathology, Medical University of Vienna. This cell line was grown in Roswell Park Memorial Institute Medium (RPMI-1640) supplemented with 10% FBS and 2 mM L-glutamine. The human hepatocellular carcinoma cell line Huh7 was kindly provided by Dr. Nataliya Rohr-Udilova, Division of Gastroenterology and Hepatology, Medical University of Vienna. Huh7 cells were grown in high-glucose DMEM with 4 mM L-glutamine, complemented with 10% FBS. 

All cells were kept in a humidified incubator (37 °C, 5% CO_2_) at the Department of Biomedical Imaging and Image-guided Therapy.

For experiments, we used either fully supplemented MEM or glucose-free DMEM supplemented with 10% fetal bovine serum (FBS). FBS has an average glucose content of 125 mg/100 mL [37]. Supplementation therefore results in an average concentration of 0.13 g/L as compared to a glucose concentration of around 1.13 g/L in fully supplemented MEM that we used for “glucose” experiments. Glucose content was therefore reduced to approximately one tenth. For starving experiments, glucose-reduced DMEM was already applied to the cells 1 or 2 h prior to 2-[^18^F]FDG addition.

### 4.3. Enzymatic In Vitro Synthesis of 2-[^18^F]FDG Radiometabolites

In general, reference radiometabolites were prepared via enzymatic in vitro synthesis on the basis of previously published procedures by Rokka et al. [18] and Kaarstadt et al. [15] (Figure 1). After centrifugation (22 °C, 13,684 *g*), the reaction mixes were analyzed with HPLC as described for the cell samples.

### 4.4. 2-[^18^F]FDG-6-P

Around 20 MBq 2-[^18^F]FDG was added to a phosphate buffered saline (PBS) solution containing 100 μL hexokinase, 2 mg adenosine triphosphate (ATP) and 1 mg magnesium chloride. The pH was adjusted to 7–7.5 with 1 M sodium hydroxide (NaOH). The mixture was mixed in a shaker (Eppendorf, Hamburg, Germany) for 2 h at 37 °C.

### 4.5. 2-[^18^F]FD-PGL

After synthesis of 2-[^18^F]FDG-6-P, 4.5 mg β-nicotinamide adenine dinucleotide phosphate and 35 units of G6PD (from yeast or leuconostoc mesenteroides) were added. The solution was again incubated at 37 °C for 2.5 h. 

### 4.6. 2-[^18^F]FDG-1,6-P_2_

In that case, the 2-[^18^F]FDG-6-P solution was further processed with 20 units PGM, 1 mg α-D-glucose-1,6-diphosphate cyclohexylammonium salt, 1 mg EDTA and 1.3 mg BSA. The pH was adjusted to around 7.9 with 1 M NaOH. The mixture was again shaken at 37 °C for 2.5 h.

### 4.7. 2-[^18^F]FDG-1-P

This metabolite was detected after 2 h, when synthesizing 2-[^18^F]FDG-1,6-P_2_ under suboptimal conditions (pH < 7.9).

### 4.8. UDP-2-[^18^F]FDG

After synthesis of 2-[^18^F]FDG-6-P, the solution was filtered with a Centrifree Ultrafiltration Device with Ultracel PL membrane (cutoff 30 kDa, Merck KGaA, Darmstadt, Germany) by centrifugation at 1520× *g* (15 min, RT) in order to remove hexokinase (~100 kDa). Then, 20 units PGM, 2 μmol UTP and 10 units UGPase were added for 3 h.

### 4.9. 2-[^18^F]FDM-6-P

As PBS can hamper the activity of phosphoglucose isomerase (PGI) according to the supplier, another batch of 2-[^18^F]FDG-6-P was produced in HEPES buffered saline solution (pH 7.5). After the formation of 2-[^18^F]FDG-6-P was confirmed, 200 units of PGI were added to synthesize 2-[^18^F]FDM-6-P. The mixture was incubated at 37 °C for another 3 h.

### 4.10. 2-[^18^F]FDG Accumulation Experiments

The workflow for accumulation experiments is shown in Figure 2. Cells were seeded into 6-well plates two days prior, reaching 80–90% confluency on the day of the experiment. For the experiment, growth medium was withdrawn and cells were washed once with the respective medium, before fully supplemented MEM or glucose-reduced DMEM were added, containing 1MBq/mL 2-[^18^F]FDG (*n* = 5). Then, 1 h after addition of 2-[^18^F]FDG, aliquots of the supernatants were taken, cells were washed two times with PBS, detached with 500 µL of Accutase and resuspended in MEM. A 100 µL aliquot of the cell suspension was measured with a gamma counter (PerkinElmer, 2480 Automatic Gamma counter, Wizard23) and subsequently, cells were mixed with trypan blue and counted with a Neubauer chamber. Values are expressed as % applied dose (% AD) per 10^4^ cells. 

The aliquots of the supernatants were filtered through a Microcon-30 kDa Centrifugal Filter Unit (Merck KGaA, Darmstadt, Germany) to remove LDH from the medium. Then, lactate concentration was determined with a lactate assay kit (Merck KGaA, Darmstadt, Germany) following the supplier’s instructions.

### 4.11. Radiometabolite Detection with HPLC

While we used one of the anion-exchanger columns applied by Rokka et al. [18] for radiometabolite separation, we used a different HPLC system and a different experimental setup. The HPLC method by Rokka et al. [18] was therefore adapted to our system for an optimal separation of the peaks by changing the gradient profile and the run time (Table S1). The final gradient profile of solvent A (0.6 M sodium dihydrogen phosphate buffer with 3% methanol) and B (3% methanol in water) was 0–12 min 5% A, 13 min 10% A, 14–18 min 15% A, 19–32 min 50% A and 34–35 min 5% A. Table S1 shows a comparison between the HPLC method by Rokka et al. [18] and our method. 

Figure 2 shows the workflow of HPLC experiments to determine intracellular radiometabolite formation. 2-[^18^F]FDG was applied under glucose conditions, glucose-reduced conditions or 1 or 2 h starving (addition of glucose-reduced DMEM prior to 2-[^18^F]FDG). In general, cells were seeded, treated and washed as described for the accumulation experiments (*n* ≥ 9 except for 2 h starve, where *n* = 7 (HT1080), 6 (Huh7) and 3 (HT29)). However, cells were not detached with Accutase, but lysed with 250 µL of methanol (MeOH) and scratched off the surface with a cell scraper. Lysates were then centrifuged for 4 min (4 °C, 13,684 *g*) and supernatants were subsequently injected into an HPLC system (Agilent Technologies, Santa Clara, CA, USA) with a radio detector (Ramona, Elysia-Raytest, Straubenhardt, Germany) and a cooled sample table. Radiometabolites were separated with an anion-exchanger Partisil™ 10 SAX column (250 mm × 4.6 mm, Supelco^®^ analytical, PA, USA) with a flow of 1 mL/min. Chromatograms were subsequently evaluated with the software GINA Star™ (Version 5.9 Service Pack 17, Ramona, Elysia-Raytest, Straubenhardt, Germany). 

The limit of detection was defined as three times and the limit of quantification as at least five times the area under the curve of the background area as described by González, O., & Alonso, R. M. [38]. The results presented in this article were corrected for the background area by manual integration of the peaks of interest. For every chromatogram the background was quantified separately.

### 4.12. Efflux Experiments

2-[^18^F]FDG was applied to the cells in either MEM or glucose-reduced DMEM as described above. Then, 1 h after 2-[^18^F]FDG addition, the supernatant was withdrawn and cells were washed three times with fresh medium, before 1.5 mL of new, non-radioactive medium (MEM or DMEM) was added carefully. After 1 h, 500 µL of the supernatant was collected, centrifuged at 13,684 *g* and injected into HPLC (*n* = 3 each). Another aliquot was measured in the gamma counter to quantify extracellular radioactivity. Values are again expressed as % AD (decay corrected). To ensure that the cell membranes were still intact after the washing steps and medium changes, aliquots of the supernatants were taken before the washing steps and at the end of the efflux experiments to determine extracellular LDH activity with an LDH assay kit (Thermo Fisher Scientific Inc., Waltham, MA, USA) according to the supplier’s instructions.

### 4.13. Statistics

#### 4.13.1. General

The values are depicted as mean ± SD. All experiments were performed in triplicate (or in duplicate in the case of radiometabolite analysis after 1 h) and repeated at least three times. Two-sided *t*-tests were performed with the software Prism 7.03 (GraphPad, San Diego, CA, USA) and *p*-values ≤ 0.05 were considered statistically significant.

#### 4.13.2. Gaussian Model

For a further analysis of the metabolic pattern, a network analysis was performed with a graphical Gaussian model as recently described [27] using JASP software (JASP Team, Version 0.14.1, https://jasp-stats.org/, accessed on 7 June 2021). This analysis uses partial correlation, i.e., the correlation between the residuals resulting from linear regression, as a measure of independence between two data points, allowing us to distinguish between direct and indirect interactions. Line thickness indicates the strength of the positive partial correlation. A detailed description of the Gaussian network model theory can also be found in the Supplementary Materials of Perišić et al. [39]. 

## 5. Conclusions

Overall, we could show that the extent of 2-[^18^F]FDG metabolism is not the same in different cancer cell lines and that glucose deprivation significantly enhances radiometabolite formation and total intracellular accumulation dependent on the cell line. Building on the publication by Kernstine K.H. et al., suggesting that 2-[^18^F]FDG PET predicts the use of glucose in pathways other than glycolysis [7], we claim that 2-[^18^F]FDG metabolism can be an important readout for the cells’ metabolic (re-)wiring. From a translational point of view and bearing in mind that 2-[^18^F]FDG radiometabolites could be an important readout for tumor heterogeneity, we need to have a closer look at the biological processes involved. Instead of simple static quantifications of 2-[^18^F]FDG uptake at single timepoints, the kinetic behavior of 2-[^18^F]FDG in each voxel over time should be assessed. New imaging technologies for whole-body PET combined with kinetic modelling and parametric analysis of the given images over time could provide an important toolset for this methodological challenge.

## Figures and Tables

**Table 1:** Mean radiometabolite formation (%) under different conditions in HT1080, HT29 and Huh7. Conditions: glucose = 1.13 g/L, glucose-reduced = 0.13 g/L, starve = glucose-reduced medium 1 or 2 h prior to 2-[^18^F]FDG addition. Radiometabolites at ~6, ~22, ~23.5 and ~27 min were excluded due to their negligible formation.

		HT1080							
Radiometabolites	RT (min)	Glucose		Glucose-Reduced		Starve (1 h)		Starve (2 h)	
		mean (%)	SD	mean (%)	SD	mean (%)	SD	mean (%)	SD
2-[^18^F]FDG	~4	**11**	3	**0.3**	0.1	**0.40**	0.09	**0.62**	0.28
2-[^18^F]FDG-1-P	~7	**0**	0	**1.6**	0.7	**3.18**	2.61	**5.7**	3.5
2-[^18^F]FDG-6-P	~9.5	**79**	2	**79**	4	**78**	9	**66**	12
2-[^18^F]FDM-6-P	~12	**0**	0	**9**	5	**6**	5	**12**	1
radiometabolite 5	~19.5	**0**	0	**0.53**	0.44	**0.56**	0.25	**2**	1
UDP-2-[^18^F]FDG	~21	**0**	0	**0.51**	0.23	**0.69**	0.40	**1.7**	0.8
2-[^18^F]FD-PGL	~25	**0.0**	0.0	**4**	2	**7**	4	**12**	7
2-[^18^F]FDG-1,6-P_2_	~28	**8**	2	**3**	2	**3**	2	**0.47**	0.11
radiometabolite 12	~29.5	**1.1**	0.6	**0.95**	0.60	**0.96**	0.82	**0.22**	0.02
		**HT29**							
**Radiometabolites**	**RT (min)**	**Glucose**		**Glucose-Reduced**		**Starve (1 h)**		**Starve (2 h)**	
		**mean (%)**	**SD**	**mean (%)**	**SD**	**mean (%)**	**SD**	**mean (%)**	**SD**
2-[^18^F]FDG	~4	**3**	1	**0.4**	0.2	**0.57**	0.14	**0.4**	0.1
2-[^18^F]FDG-1-P	~7	**1.2**	0.5	**3**	1	**3**	2	**1.2**	0.2
2-[^18^F]FDG-6-P	~9.5	**79**	4	**59**	9	**56**	10	**69.0**	0.7
2-[^18^F]FDM-6-P	~12	**7**	2	**14**	3	**17**	3	**19**	1
radiometabolite 5	~19.5	**0.1**	0.1	**1.5**	0.9	**2.9**	1.0	**2.2**	0.4
UDP-2-[^18^F]FDG	~21	**0.1**	0.2	**1.0**	0.6	**2.1**	0.5	**2.0**	0.3
2-[^18^F]FD-PGL	~25	**3**	1	**15**	7	**15**	8	**5**	2
2-[^18^F]FDG-1,6-P_2_	~28	**4**	3	**3**	3	**1**	1	**0.3**	0.1
radiometabolite 12	~29.5	**3**	1	**3**	1	**2**	1	**0.7**	0.2
		**Huh7**							
**Radiometabolites**	**RT (min)**	**Glucose**		**Glucose-reduced**		**Starve (1 h)**		**Starve (2 h)**	
		**mean (%)**	**SD**	**mean (%)**	**SD**	**mean (%)**	**SD**	**mean (%)**	**SD**
2-[^18^F]FDG	~4	**8**	2	**2**	1	**3**	2	**2**	1
2-[^18^F]FDG-1-P	~7	**0.7**	0.4	**4**	2	**3**	2	**4**	2
2-[^18^F]FDG-6-P	~9.5	**86**	3	**76**	6	**76**	5	**77**	6
2-[^18^F]FDM-6-P	~12	**4**	2	**13**	4	**10**	6	**8**	5
radiometabolite 5	~19.5	**0.2**	0.3	**0.5**	0.3	**1.5**	0.5	**2**	1
UDP-2-[^18^F]FDG	~21	**0.1**	0.2	**1.1**	0.5	**1.8**	0.7	**2.7**	1.0
2-[^18^F]FD-PGL	~25	**0.4**	0.8	**3**	1	**3.4**	0.8	**3**	2
2-[^18^F]FDG-1,6-P_2_	~28	**0.7**	0.3	**0.2**	0.2	**0.1**	0.1	**0.4**	0.1
radiometabolite 12	~29.5	**0.2**	0.2	**0.1**	0.2	**0.06**	0.07	**0.2**	0.2

## Data Availability

Data is contained within the article and supplementary files.

## Supplementary Materials

The following are available online at https://www.mdpi.com/article/10.3390/ph14090910/s1, Figure S1: LDH activity in the supernatants of HT1080 and HT29, 1 h after changing the medium., Table S1: Comparison between the HPLC method of Rokka et al. [18] and our adapted method.
