# Supplementary material for: If It Works, Don’t Touch It? A Cell-Based Approach to Studying 2-[18F]FDG Metabolism
Source: Pharmaceuticals (Basel). 2021 Sep 9;14(9):910. doi: 10.3390/ph14090910 (PMC8467898; doi:10.3390/ph14090910)
Supplement: Supplementary file 1 [file pharmaceuticals-14-00910-s001.zip › pharmaceuticals-1342485-supplementary.pdf]

Supplementary materials

Table S1. Comparison between the HPLC method of Rokka et al. [18] and our adapted method.

**Rokka et al.**			**Our method**		
**HPLC system:** Merck Hitachi			**HPLC system:** Agilent technologies		
**Columns:** Radial-Pak 8P SAX 10 µm (100 mm × 8 mm) or Partisil SAX 10 µm (250 mm × 4.6 mm)			**Column:** Partisil SAX 10 µm (250 mm × 4.6 mm)		
**Run time:** 30 min			**Run time:** 35 min		
**Flow:** 1 mL/min			**Flow:** 1 mL/min		
**Solvents:** A = 0.6 M sodium dihydrogen phosphate buffer with 3% methanol; B = 3% methanol in water			**Solvents:** A = 0.6 M sodium dihydrogen phosphate buffer with 3% methanol; B = 3% methanol in water		
**Gradient profile:**					
**Time (min)**	**%A**	**%B**	**Time (min)**	**%A**	**%B**
0-10	5	95	0-12	5	95
11-18	15	85	13	10	90
19-29	50	50	14-18	15	85
30	5	95	19-32	50	50
			34-35	5	95

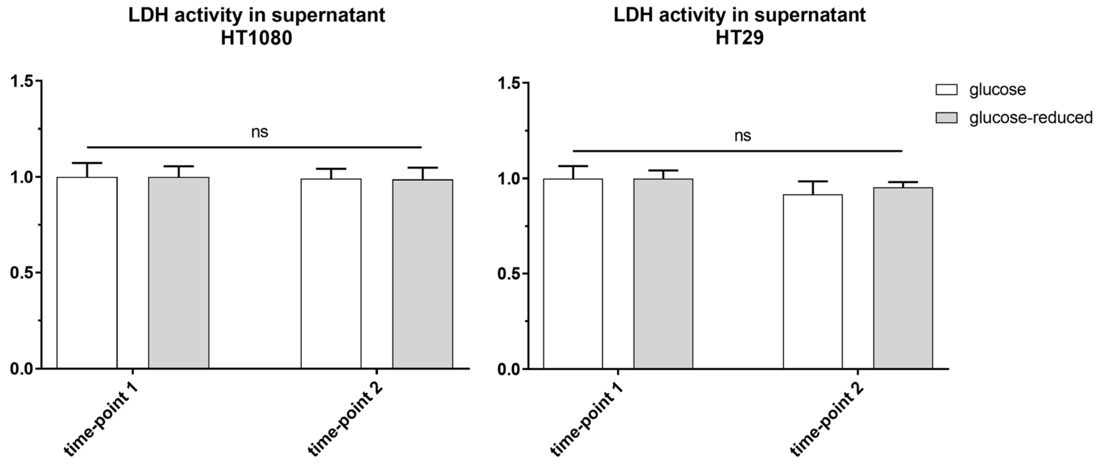

Figure S1. LDH activity in the supernatants of HT1080 and HT29, 1 h after changing the medium. Conditions: glucose = 1.13 g/L, glucose-reduced = 0.13 g/L.
